# Soil pH Is the Primary Factor Correlating With Soil Microbiome in Karst Rocky Desertification Regions in the Wushan County, Chongqing, China

**DOI:** 10.3389/fmicb.2018.01027

**Published:** 2018-05-29

**Authors:** Daihua Qi, Xuwen Wieneke, Jianping Tao, Xu Zhou, Udaya Desilva

**Affiliations:** ^1^Key Laboratory of Eco-Environments in Three Gorges Reservoir Region (Ministry of Education), Chongqing Key Laboratory of Plant Ecology and Resources Research in Three Gorges Reservoir Region, School of Life Sciences, Southwest University, Chongqing, China; ^2^Department of Animal Science, Oklahoma State University, Stillwater, OK, United States

**Keywords:** karst rocky desertification (KRD), soil bacteria, soil bacterial community, soil properties, driving factors, Illumina HiSeq sequencing

## Abstract

Karst rocky desertification (KRD) is a process of land degradation, which causes desert-like landscapes, deconstruction of endemic biomass, and declined soil quality. The relationship of KRD progression with above-ground communities (e.g. vegetation and animal) is well-studied. Interaction of soil desertification with underground communities, such as soil microbiome, however, is vastly unknown. This study characterizes change in soil bacterial community in response to KRD progression. Soil bacterial communities were surveyed by deep sequencing of 16S amplicons. Eight soil properties, pH, soil organic matter (SOM), total and available nitrogen (TN and AN), total and available phosphorus (TP and AP), and total and available potassium (TK and AK), were measured to assess soil quality. We find that the overall soil quality decreases along with KRD progressive gradient. Soil bacterial community compositions are distinguishingly different in KRD stages. The richness and diversity in bacterial community do not significantly change with KRD progression although a slight increase in diversity was observed. A slight decrease in richness was seen in SKRD areas. Soil pH primarily correlates with bacterial community composition. We identified a core microbiome for KRD soils consisting of; Acidobacteria, Alpha-Proteobacteria, Planctomycetes, Beta-Proteobacteria, Actinobacteria, Firmicutes, Delta-Proteobacteria, Chloroflexi, Bacteroidetes, Nitrospirae, and Gemmatimonadetes in this study. Phylum Cyanobacteria is significantly abundant in non-degraded soils, suggesting that Cyanobacterial activities might be correlated to soil quality. Our results suggest that Proteobacteria are sensitive to changes in soil properties caused by the KRD progression. Alpha- and beta-Proteobacteria significantly predominated in SKRD compared to NKRD, suggesting that Proteobacteria, along with many others in the core microbiome (Acidobacteria, Actinobacteria, Firmicutes, and Nitrospirae), were active in nutrient limiting degraded soils. This study demonstrates the relationship of soil properties with bacterial community in KRD areas. Our results fill the gap of knowledge on change in soil bacterial community during KRD progression.

## Introduction

Karst is a type of natural landscape with a fragile ecosystem. Land in karst area is highly susceptible to environmental disturbances (Calò and Parise, 2006). Soil structure in the karst region is unstable and prone to deterioration (Gutiérrez et al., 2014). Karst rocky desertification (KRD) is the progress of soil desertification. This kind of desertification often occurs in the karst area, resulting in desert-like landscape (Yan and Cai, 2015). Increased soil erosion and bedrock exposure along with decreased soil quality and biomass are main consequences of KRD progression (Ford and Williams, 1989). Decreased soil quality is defined as low biological productivity, environmental quality, and plant/animal health (Karlen et al., 1997). Severe soil desertification deprives cultivated land and affects global geomorphology (Jiang et al., 2014). Altogether, the progression of KRD has been drawing considerable attention. Karst landscape widely exists in southwest of China, especially in Yunan, Guizhou, and Chongqing (Peng et al., 2011). Karst area is classified into no-, light-, moderate-, and severe-KRD (NKRD, LKRD, MKRD, and SKRD); based on bedrock exposure rate, vegetation, and soil cover rate (Li et al., 2009). The land in the Wushan County (northeast Chongqing) is highly susceptible to desertification. Roughly 70% of soils (2066 km^2^) in the Wushan County are M- and S-KRD areas (Qi et al., 2017).

Many studies report causes of KRD and changes in soil and vegetation community in response to KRD progression (Calò and Parise, 2006; Xie et al., 2015; Zhang et al., 2016; Qi et al., 2017). It is suggested that besides the natural susceptibility to desertification in the karst region, anthropogenic disturbances and soil exploitation are the other main reasons accelerating soil desertification (Yan and Cai, 2015). Furthermore, soil pH significantly increases along the KRD gradient, and is associated with decreased soil quality (Wang et al., 2004). As the result, vegetation diversity and coverage significantly decreases in accordance to desertification progression (Zeng et al., 2007; Qi et al., 2017). Many studies focus on determining correlations of aboveground factors with KRD progression. Few studies, however, aim to understand change in underground community, such as soil microbiome, influenced by the KRD progression.

Soil microbiome is highly heterogeneous. Microbiome includes all the microorganisms that belong to bacteria, fungi, and archaea. Soil microbiome, therefore, is complex in structure and enormous in number. More importantly, soil microbiome has large impact on plant health, soil productivity, and ecosystem (Chaparro et al., 2012; Zolla et al., 2013; Lakshmanan et al., 2014). Soil bacteria play an essential role in soil weathering and nutrient cycling (Uroz et al., 2011). For instance, potassium solubilizing bacteria (KSB) produce low molecular weight organic acids that result in rock weathering; while other bacteria such as ammonia oxidizing bacteria (AOB) and nitrite oxidizing bacteria (NOB), produce enzymes that oxidize nitrogen compounds, thereby contributing to nutrient cycling (De Boer and Kowalchuk, 2001; Kowalchuk and Stephen, 2001; Mursyida et al., 2015). Copiotrophic bacteria, such as Firmicutes, Proteobacteria, and Actinobacteria, usually claim the predominance in fertile soils (Yao et al., 2000; Fierer et al., 2007). Oligotrophic taxa, such as Acidobacteria, on the other hand, are abundant in barren soils (Shah et al., 2011; Rime et al., 2015). Soil bacteria are particularly essential in terrestrial ecosystem, because (1) terrestrial soil is usually not supplemented with external fertilizer and (2) soil bacterial activities and byproducts affect soil fertility in the terrestrial ecosystem. It is important to characterize change in soil microbiome in response to KRD progression. Establishing changes in microbiome as well as the core microbiome helps to understand underground community composition and microbial contributions on the ecosystem in KRD areas.

In this study, we characterize change in soil bacterial community in accordance to KRD progression. Soil bacterial community was surveyed by deep sequencing of 16S hypervariable 4-5 region (V4-5) and Illumina HiSeq platform. Eight soil properties, pH, soil organic matter (SOM), total and available nitrogen (TN and AN), total and available phosphorus (TP and AP), and total and available potassium (TK and AK), were measured for soil quality. We hypothesized that (1) the composition of soil bacterial community changed along the KRD gradient; (2) changes in soil properties caused by desertification impacted on soil bacterial community; and (3) certain bacteria were sensitive to KRD progression. To address our hypotheses, we (1) surveyed soil bacterial community and identified core microbiome in KRD regions; (2) determined soil property as the primary influential factor shaping bacterial community; and (3) analyzed correlation of soil properties with core phyla found in this study.

## Materials and methods

### Study area

Detailed descriptions of study area are reported in a previous study (Qi et al., 2017). Briefly, the study area is located in the Wushan County, northeast Chongqing (N 30°46′-31°28′, E 109°33′-110°11′). The main soil types in study area are Xanthic Ferralsols and Haplic Luvisols. Soil types are classified according to guidelines of Food and Agriculture Organization of the United Nations (FAO) (Gong, 2001). Xanthic Ferralsols is generally found in the hilly and low-mid mountain zone that is below 1,500 m of elevation. The Haplic Luvisols is mainly found in degraded soils with anticlinal structure and is neutral to slightly alkaline soil pH. The bedrock in study site is composed of limestone, clastic carbonate, and dolomite.

Study sites were evaluated and classified into four groups, no KRD (NKRD), latent KRD (LKRD), moderate KRD (MKRD), and severe KRD (SKRD), based on vegetation coverage rate and bedrock exposure rate (Table 1). A total of 12 sampling quadrats (20 × 20 m) were chosen, three for each stage of KRD. Three sub-sampling quadrats (10 × 10 m) were uniformly chosen within each sampling quadrat in order to study soil microbiome community comprehensively.

**Table 1:** Features and evaluations of karst rocky desertification areas.

**Study sites**	**NKRD**	**LKRD**	**MKRD**	**SKRD**
Coordinates	N 31°8′4″ E 110°0′45″ N 31°9′1″ E 110°1′31″ N 31°8′48″ E 110°1′40″ N 31°7′54″ E 110°0′6″ N 31°2′7″ E 110°44′11″	N 31°3′32″ E 109°45′56″ N 31°4′45″ E 109°54′28″ N 31°0′4″ E 109°51′8″ N 31°0′3″ E 109°51′22″ N 31°4′21″ E 109°48′59″	N 31°50′56″ E 110°2′41″ N 31°0′1″ E 109°50′56″ N 31°0′26″ E 109°52′16″ N 31°9′30″ E 109°46′14″ N 31°7′27″ E 109°45′24″	N 31°4′42″ E 109°54′25″ N 31°2′20″ E 109°44′56″ N 31°0′15″ E 109°51′53″ N 31°1′55″ E 109°54′6″ N 31°6′49″ E 109°44′34″
Elevation (M ASL)	850–1,100	750–1,100	850–1,200	900–1,200
Vegetation	Coniferous forest	Shrub-land	Shrub-grassland	Shrub-grassland
Vegetation Coverage (%)	≥80	≥80	40–60	15–30
Bedrock	Limestone	Limestone	Limestone	Limestone
Bedrock Exposure Rate (%)	0	0–30	50–70	>70
Soil Type	Xanthic Ferralsols	Haplic Luvisols	Haplic Luvisols	Haplic Luvisols
Soil Depth (Cm)	40–70	20–40	20–30	10–20

*Geographic features, soil type, bedrock exposure rate, and vegetation coverage are summarized in the table. Classification of KRD is based on vegetation coverage, soil depth, and bedrock exposure rates. No KRD (NKRD), latent KRD (LKRD), moderate KRD (MKRD), and severe KRD (SKRD)*.

### Soil sample collection

Soil samples were collected in N-, L-, M-, and S-KRD regions with little human interference (i.e., land with no agricultural activities, vegetation restoration, or soil exploitation etc.). All the collecting tools were autoclaved. Three soil samples (5 g) were randomly and uniformly collected from the top soil layer (0–10 cm) in each sub-sampling quadrat. Any plant residues, such as roots and leaves, were either carefully avoided or removed during soil sample collection. Soil samples were collected and stored in 50 mL sterile tubes on ice (approximately 0°C). Three samples from one sub-sampling quadrat were pooled into the same 50 mL tube (15 g) to comprehensively represent soil microbiome community in a quadrat. All pooled samples from sampling quadrats were labeled as NKRD_1, NKRD_2, NKRD_3, LKRD_1, LKRD_2, LKRD_3, MKRD_1, MKRD_2, MKRD_3, SKRD_1, SKRD_2, and SKRD_3. Once arrived in the lab, soil samples were immediately mixed by Large Capacity Mixer (Glas-Col, Terre Haute, IN, USA) at 1,600 rpm, 4°C for 30 min. Two sets of soil subsamples (2 g) were collected in a sterile environment. One set of samples was sent to the Key Laboratory of Eco-environments in Three Gorges Reservoir Region for the soil properties measurement. The other set of samples was used for DNA isolation.

### Soil properties measurement

Eight soil properties were measured in this study, namely pH, soil organic matter (SOM), total and available nitrogen (TN and AN), total and available phosphorus (TP and AP), and total and available potassium (TK and AK). Detailed protocols were reported in previous studies (Li and Shao, 2006; Qi et al., 2017). Briefly, soil pH was measured using a pH meter (FE20, Mettler-Toledo Instruments, China) with a soil-to-water ratio of 1:2.5 (w/v). Soil organic carbon (SOC) was measured using potassium dichromate oxidation (Nelson and Sommers, 1982). Soil organic matter was calculated by multiplying SOC values by 1.724 (Walkley and Black, 1934). TN, AN, TK, AK, TP, and AP were measured using LAQUAtwin ion meter kits (Horiba Instruments, Singapore).

### DNA isolation

DNA was isolated by using the MoBio PowerSoil DNA extraction kit and following manufacturer's instructions (MoBio Laboratories, Carlsbad, CA, USA). A total of 0.5 g sub-sampled soil was used. In order to prevent DNA shearing, an additional incubation step was added for 10 min at 65°C, followed by bead beating for 2 min (Lauber et al., 2009). DNA samples were investigated by using a 0.8% (wt/vol) low melting point agarose gel. High quality DNA was isolated and purified by using an agarose gel DNA purification kit (TaKaRa Bio USA, Inc., Mountain View, CA, USA). Purified DNA samples were quantified by using NanoDrop ND-1000 spectrophotometer (NanoDrop Technologies, Wilmington, DE, USA). Samples were stored at −20°C till use.

### PCR amplification and 16S V4-5 region sequencing

An aliquot of 50 ng DNA template was used for PCR amplification. The 16S V4-5 region was amplified using primer set: 515F (5′-GTGCCAGCMGCCGCGG-3′) and 907R (5′-CCGTCAATTCMTTTRAGTTT-3′) with barcodes. PCR amplification was performed by using Phusion® High-Fidelity PCR Master Mix (New England Biolabs, Ipswich, MA, USA). Quantification and qualification of PCR products were performed by mixing the same amount of 1X loading buffer that contained SYBR green with PCR products. Samples were loaded on 2% electrophoresis agarose gel for detection. Gel purification was performed using Qiagen Gel Extraction Kit (Qiagen, Hilden, Germany).

TruSeq DNA PCR-Free Sample Preparation Kit (Illumina, San Diego, CA, USA) was used to conduct sequencing library by following manufacturer's recommendations. Index codes were then added. The library quality was verified using the Qubit 2.0 Fluorometer (Thermo Scientific, Waltham, MA, USA) and Agilent Bioanalyzer 2100 system. The library was sequenced on an Illumina HiSeq2500 instrument (Illumina, Inc., San Diego, CA, USA), 250 bp paired-end raw reads were generated.

### Bioinformatic analysis

Mothur (v. 1.39.5) was used for data mining following mothur MiSeq standard operating procedure (Schloss et al., 2009). Briefly, “make.contigs” command was used to assemble paired-end reads. Barcode and primer sequences were removed. Command “screen.seqs” was used to trim the data with the criteria “maxambig = 0, maxhomop = 8, and maxlength = 380.” Contigs that were longer than 380 bp, or contained longer than 8 homopolymers, or contained undermined base were excluded. “trim.seqs,” “pre.cluster,” and “chimera.uchime,” were used to further process sequences. Uchime was used to remove chimeric sequences (Edgar et al., 2011). Sequences were then classified using the Bayesian classifier with the “classif.seqs” command. Silva non-redundant v123 database was used as the reference (Quast et al., 2013). Archaea, chloroplasts, and mitochondria were excluded by using command “remove.lineage” with criteria “taxon = Chloroplast-Mitochondria-unknown-Archaea-Eukaryota.” Command “cluster.split” was used to assign qualified sequences into operational taxonomic units (OTUs) based on at least 97% similarity (OTU_0.03_). Command “classify.otu” was used to classify all the sequences of OTU_0.03_ into taxonomic groups at the bootstrap threshold of 80%. Random sub-sampling with the least amount of sequences among all the samples was conducted to avoid sequencing bias. The similarities among communities based on communities' memberships were measured by using unifrac-based metrics generated with command “unifrac.weighted” (Lozupone et al., 2011). Raw sequencing data was submitted to Sequence Read Archive (SRA) in NCBI, access number SRP124255.

### Statistical analysis

One-way analysis of variance (One-way ANOVA) and Pearson correlation analysis were performed by using R 3.3.2 statistical software (Team, 2013). Tukey's (honest significant difference) HSD test was used to determine differences of soil properties caused by soil degradation. The change in soil properties were considered significantly different if *P* < 0.05. Correlations of soil properties with microbiome were determined by using Pearson correlation analysis. Soil properties and microbiome were considered significantly correlated if the *P* < 0.05.

Linear discriminant analysis (LDA) Effect Size (LEfSe) was performed to determine the soil microbiome that significantly responded to KRD progression (Segata et al., 2011). More specifically, the non-parametric factorial Kruskal-Wallis (KW) sum-rank test was first used to detect microbiome with significant abundant differences between KRD stages (*P* < 0.05). The unpaired Wilcoxon rank-sum test was used to compare the significant abundant differences among taxa under influences of soil desertification (*P* < 0.05). Linear Discriminant Analysis was applied to calculate the effective size of abundant differences. The LAD scores were normalized by log10.

Distance-based redundancy analysis (db-RDA) was conducted by using vegan package in R (Oksanen et al., 2014). The db-RDA was used to perform the direct gradient analysis. Constrains and accumulates explanatory variables are scattered on the ordination exes (RDA1 and RDA2). The two ordinations axes (RDA1 and RDA2) were constructed by constraining an entire set of explanatory variables, eight soil properties in this study. Mantel test at 999 permutations was used to determine impact of soil properties on bacterial community composition. Impact of soil properties on microbiome community was considered significant if *P* < 0.05. Weighted unifrac-distance was used to determine soil bacterial community structural similarity. Communities with similar compositions closely clustered, and vice versa.

The multivariate regression tree (MRT) was conducted by using the mvpart package in R (De'Ath, 2002). The MRT tree was used to reveal the relationship of relative abundance of core phyla with changes in soil properties caused by KRD progression.

## Results

### Change in soil properties along with desertification progression

**Figure 1:**
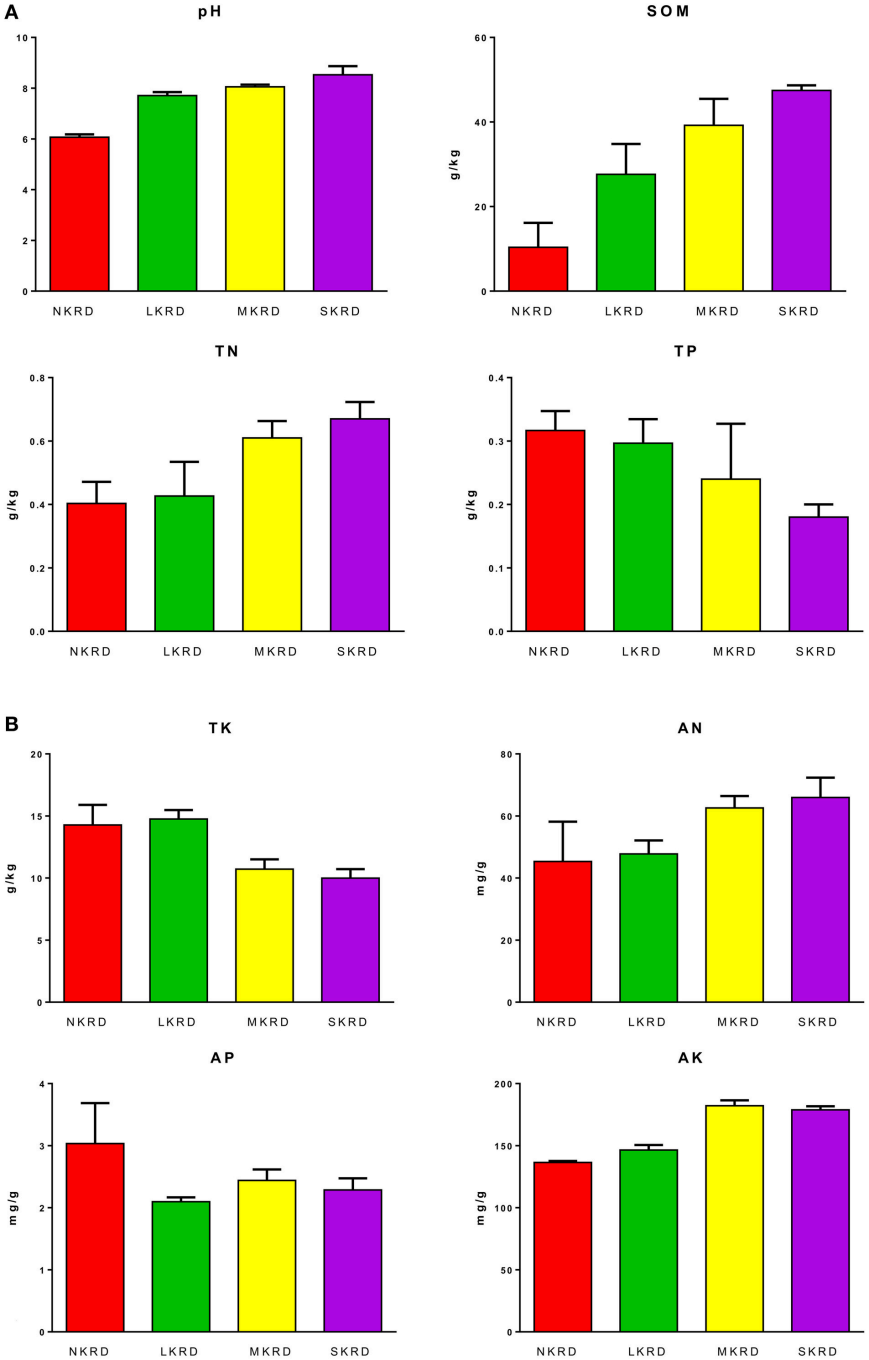
Change in soil properties along with karst rocky desertification gradient **(A,B)**. No KRD (NKRD) is in red color, latent KRD (LKRD) is in green color, moderate KRD (MKRD) is in yellow color, and severe KRD (SKRD) is in purple color. Soil organic matter (SOM), total and available nitrogen (TN and AN), total and available phosphorus (TP and AP), and total and available potassium (TK and AK).

Changing trends of soil properties in KRD regions are shown in Figures 1A,B. Detailed values of soil properties are shown in Supplementary Table 1. One-way ANOVA (Supplementary Table 2) followed by Tukey's HSD test (Table 2) were used to determine differences of soil properties among KRD areas. Overall, soil pH, SOM, and AK significantly increased along with degradation gradients (*P* < 0.05); whereas TK significantly decreased (*P* = 0.03). Total nitrogen in M- and S-KRD areas was significantly higher compared to that of in N- and L-KRD regions; whereas TP in SKRD was significantly lower than that of in NKRD regions.

**Table 2:** Average value of soil properties in karst rocky desertification regions.

	**pH**	**SOM g/kg**	**TN g/kg**	**TP g/kg**	**TK g/kg**	**AN mg/g**	**AP mg/g**	**AK mg/g**
NKRD	6.07c	10.36b	0.40b	0.32a	14.28ab	45.36a	3.03a	136.46b
LKRD	7.71b	27.63b	0.43ab	0.30ab	14.75a	47.77a	2.10a	146.44b
MKRD	8.05b	39.22ab	0.61a	0.24ab	10.72b	62.61a	2.44a	182.17a
SKRD	8.53a	47.47a	0.67a	0.18b	9.99b	65.97a	2.29a	178.89a

*The Tukey's HSD test is used to determine the significant difference between variables. Different letters are assigned to significant difference, P < 0.05. No KRD (NKRD), latent KRD (LKRD), moderate KRD (MKRD), and severe KRD (SKRD). Soil organic matter (SOM), total and available nitrogen (TN and AN), total and available phosphorus (TP and AP), and total and available potassium (TK and AK)*.

### Soil microbiome in karst rocky desertification regions

Total of 12 soil samples were sequenced by targeting 16S V4-5 region, with three replicates in each KRD area. Soil samples were surveyed to study change in soil bacterial community along with karst desertification progression. A total of 1,340,457 paired-end raw reads were generated using Illumina HiSeq platform. Averages of 111,704 ± 28,034 paired-end raw reads were obtained per sample. A total of 1,181,535 qualified reads remained after data trimming and sub-sampling, with an average of 98,461 ± 25,371 reads per sample. A total of 25 phyla were classified with qualified reads. All the reads were further categorized at class, order, family, and genus levels. Overall, 54 classes, 116 orders, 205 families, and 487 genera were classified among all the samples. Detailed OTU information is shown in Supplementary Table 3.

Twelve phyla took up more than 1% of the total abundance, namely Acidobacteria (24.17%), Alpha-Proteobacteria (20.57%), Planctomycetes (11.51%), Beta-Proteobacteria (9.39%), Actinobacteria (6.90%), Firmicutes (5.97%), Deltaproteobacteria (4.80%), Chloroflexi (4.73%), Bacteroidetes (4.43%), Nitrospirae (2.13%), Gemmatimonadetes (1.29%), and Deinococcus-Thermus (1.26%). Core microbiome at phylum level was defined as phyla that were found in all 15 samples and took up at least 1% of the relative abundance within a sample. Under such criteria, phyla Acidobacteria, Alpha-Proteobacteria, Planctomycetes, Beta-Proteobacteria, Actinobacteria, Firmicutes, Deltaproteobacteria, Chloroflexi, Bacteroidetes, Nitrospirae, and Gemmatimonadetes were the core microbiome in this study. Out of all the core phyla, Alpha- and Beta-Proteobacteria were responsive to changes in soil properties and significantly predominated in SKRD compared to those of in NKRD (Supplementary Figure 1). The one-way ANOVA followed by Tukey's HSD test are shown in Supplementary Tables 4, 5.

Sixteen genera took up more than 1% of the total reads, namely *Halomonas* (32.10%), *Aliihoeflea* (5.75%), *Pelagibacterium* (4.89%), *Pseudomonas* (4.84%), *Gemmata* (3.56%), *Nitrospira* (3.13%), *Lysobacter* (2.65%), *Nesterenkonia* (2.57%), *Methylocystis* (2.11%), *Meiothermus* (1.82%), *Ramlibacter* (1.66%), *Lactobacillus* (1.56%), *Methylophilus* (1.34%), *Bryobacter* (1.30%), *Bacillus* (1.14%), and *Burkholderia* (1.04%). Core microbiome at genus level is defined as genera that existed in all the samples with at least 1% of the relative abundance within a sample. Genera *Halomonas, Aliihoeflea, Pelagibacterium, Pseudomonas, Gemmata, Nitrospira, Nesterenkonia, Methylocystis, Meiothermus, Ramlibacter, Lactobacillus, Methylophilus, Bryobacter*, and *Bacillus* were core genera in this study. Many of the core phyla had functions involved with nutrient cycling and mineral weathering in soil, which might contribute to change in soil properties.

Lefse analysis was used to identify phylum and genus that significantly responded to KRD progression (**Figures 3A,B**). Phylum Cyanobacteria was significantly more abundant in NKRD compared to that of in degraded soils. Genera that significantly predominated in each KRD region were determined via Lefse analysis (**Figure 3A**). Genera *Aquicella, Rhizomicrobuim, Inquillnus, Nitrobacter, Telmatobacter, Mucilaginibacter, Anaeromyxobacter*, and *Rudaea* were significantly abundant in NKRD areas; *Roseiflexus* predominated in LKRD regions; *Pseudomonas, Lysobacter, Ramlibacter, Solitalea, Flavihumibacter, Microvirga, Chryseolinea, Cupriavidus, Sphingomonas, Adaeribacter, Pseudonocardia*, and *Roseiflexus* predominated in MKRD areas; and *Rhizohium, Zavarzinella, Terrimonas, Blastocatella, Altererythrobacter, Frankia, Agromyces, Actinophytocola, Senegalimassilia*, and *Niastella* were significantly abundant in SKRD regions (**Figure 3B**).

### Bacterial community composition in karst rocky desertification regions

The Shannon and Ace indices were calculated to assess diversity and richness of soil microbiome, respectively (Supplementary Figure 2). The overall change in diversity and richness were not significantly different along with KRD gradient. The diversity slightly increased; whereas richness slightly decreased in SKRD areas.

**Figure 2:**
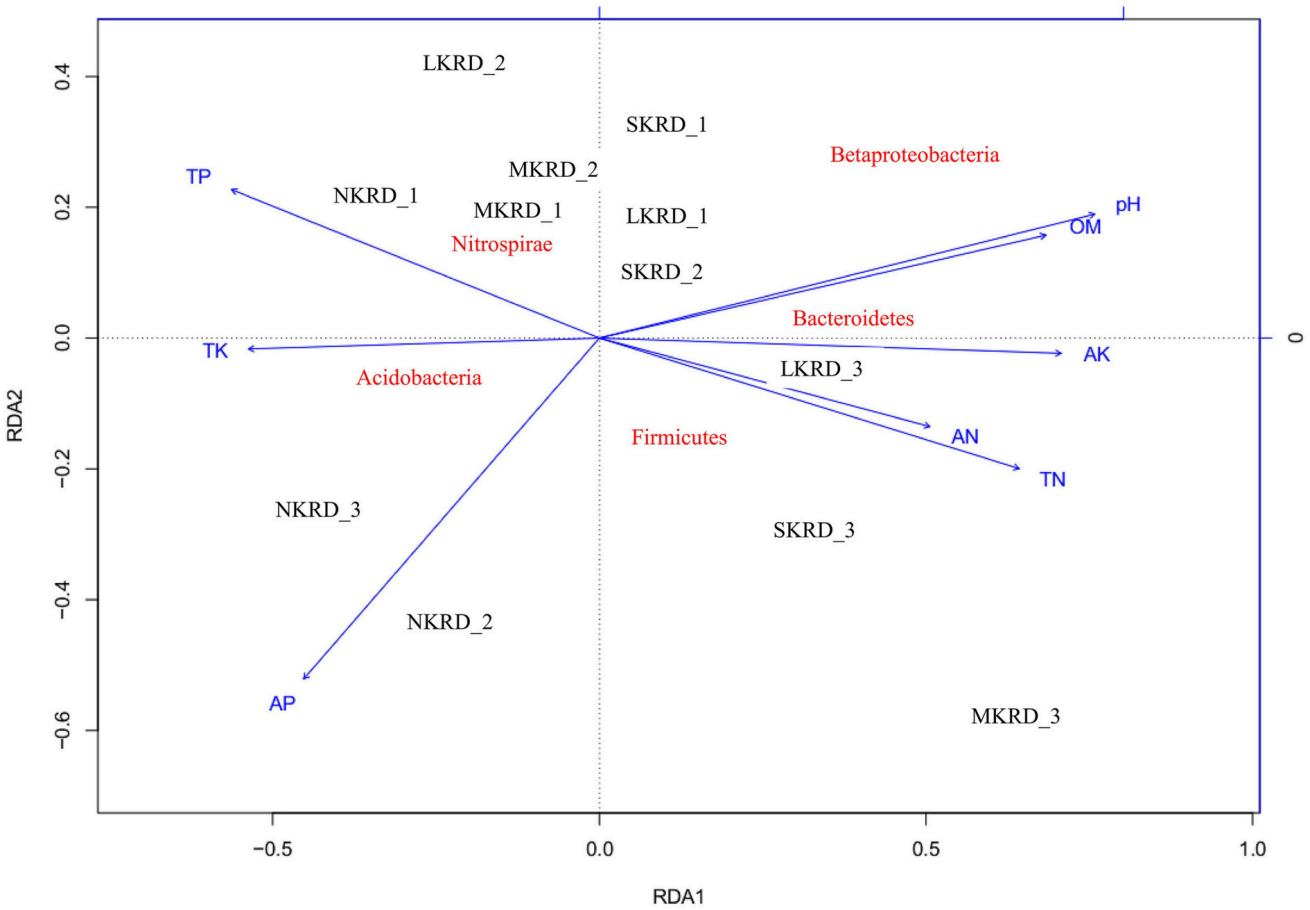
The distance-based redundancy analysis (db-RDA) diagram shows distribution of soil bacterial communities in karst rocky desertification areas. Study sites are labeled in black No KRD (NKRD), latent KRD (LKRD), moderate KRD (MKRD), and severe KRD (SKRD). Soil properties are labeled in blue. Soil organic matter (SOM), total and available nitrogen (TN and AN), total and available phosphorus (TP and AP), and total and available potassium (TK and AK). Phylum is labeled in red.

The db-RDA was used to present distribution of soil microbiome (β-diversity was calculated with unifrac-weighted matrix at phylum level) along with KRD progression (Figure 2). Result showed that soil microbiome in SKRD areas separated from the rest of communities along RDA1 axis, suggesting a distinguishable difference in bacterial community composition in SKRD soils compared to those of in other KRD areas. Soil microbiome in NKRD soils further separated from those of in L- and M-KRD regions along RDA2 axis, suggesting structural differences of microbiome between non-degraded and degraded soils.

**Figure 3:**
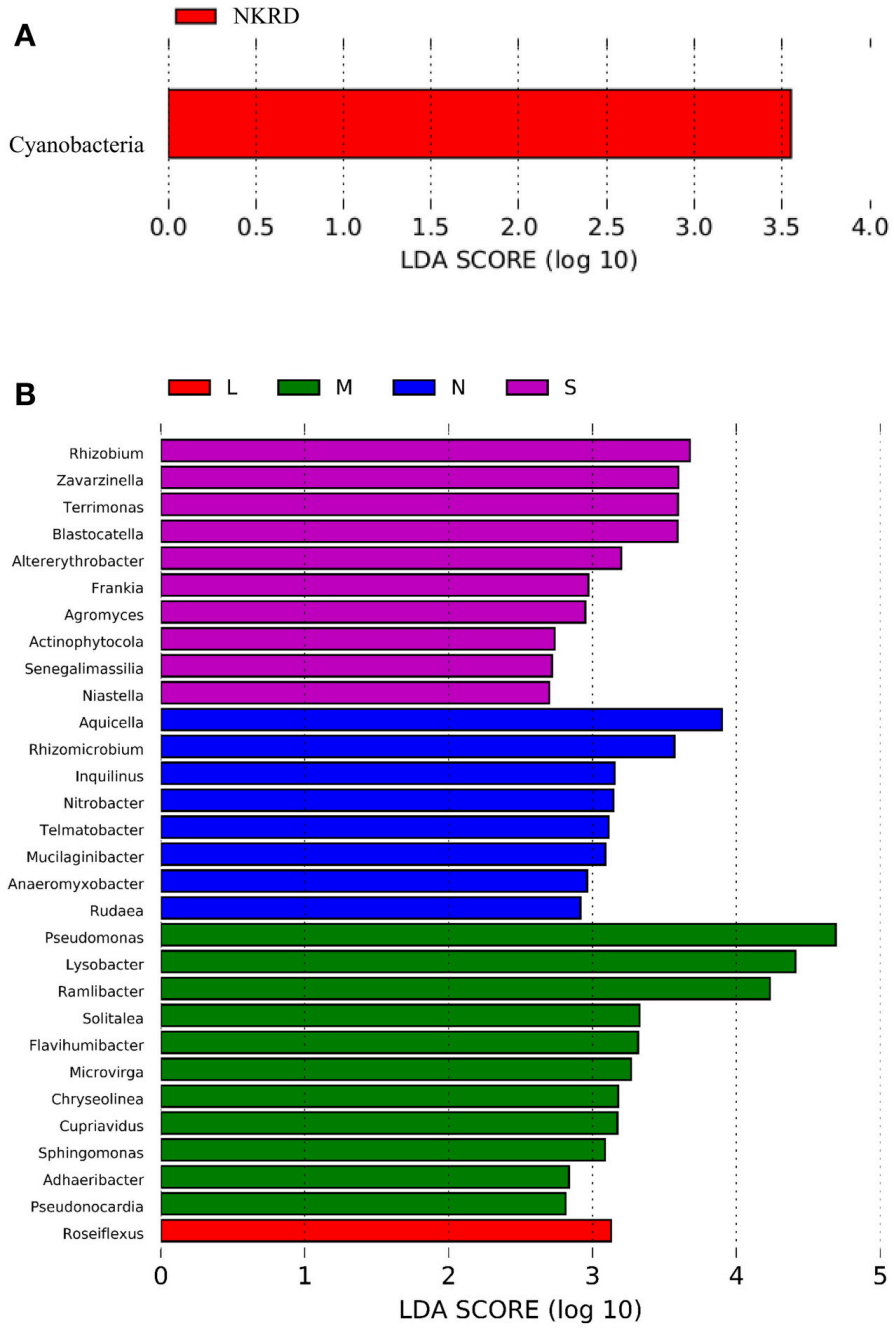
The Linear discriminant analysis (LDA) Effect Size (LEfSe) analysis identifies phylum **(A)** and genus **(B)** that respond significantly to karst rocky desertification progression. Relative abundance is significant when *P* < 0.05, logarithmic LDA score ≥2.0. No KRD (NKRD).

**Figure 4:**
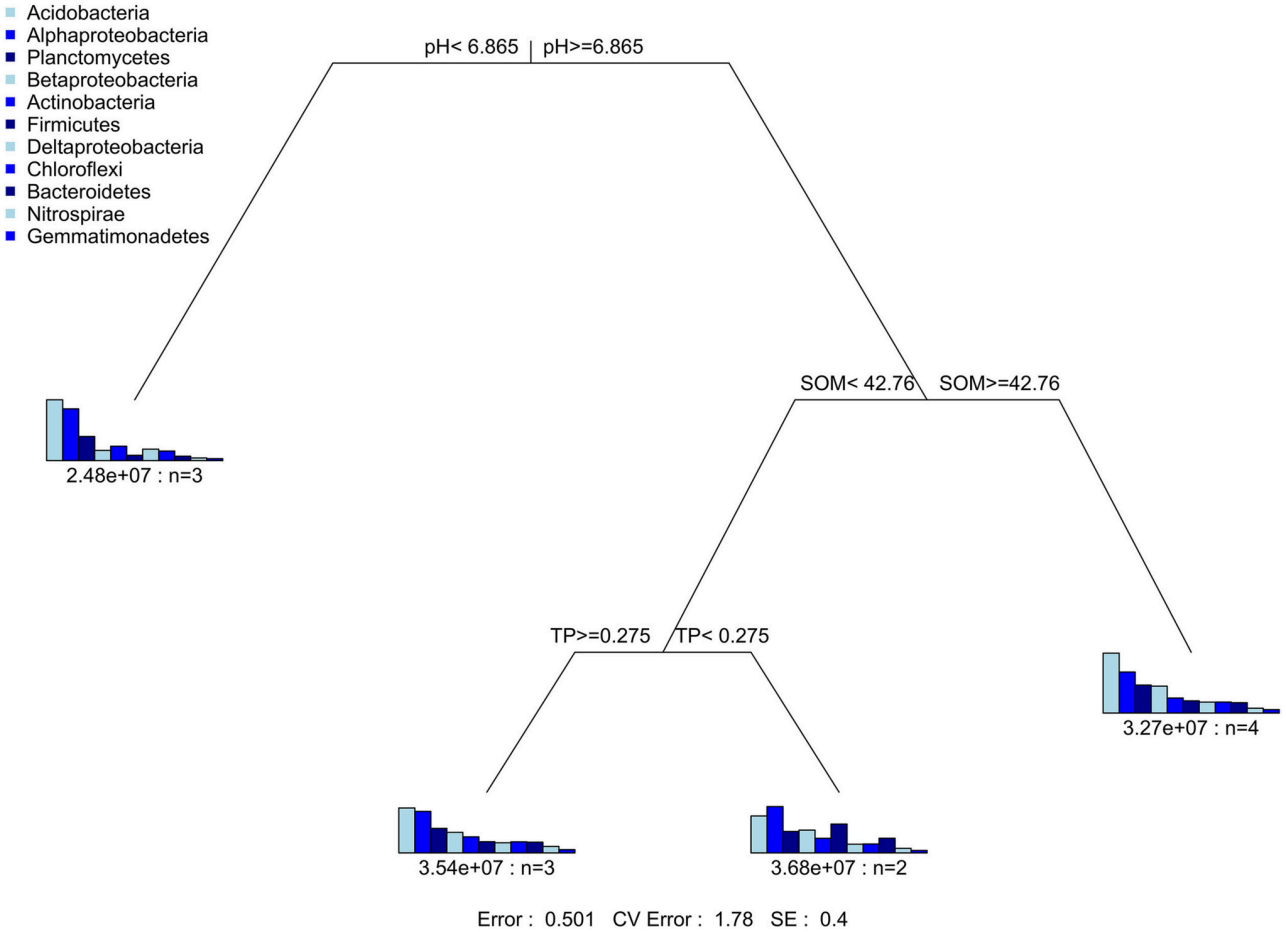
The multivariate regression tree (MRT) is used to reveal the relationship of relative abundance of core phyla with change in soil properties caused by KRD progression.

Mantel test at 999 permutations was used to calculate impact of soil properties on bacterial community compositions across desertification gradient (Table 3). Overall, pH (*r*^2^ = 0.27, *P* = 0.02) was the primary factor shaping soil microbiome community in KRD regions. The MRT analysis (Figure 4) was used to interpret the relationship of relative abundance of core phylum with changes in soil properties along with KRD progression. Results indicated that soil pH was the primary factor in explaining the variation in bacterial community compositions during KRD progression. The core phyla clustered separately in soils with pH < 6.9, which only existed in NKRD regions. The MRT analysis further verified that bacterial community was mainly impacted by soil pH.

**Table 3:** Mantel test to determine correlation of soil properties with bacterial community composition.

**Soil property**	***R*^2^**	***F***	***P-*value**
pH	0.27	3.6	0.02^*^
AK	0.07	0.89	0.49
AP	0.05	0.60	0.63
TK	0.03	0.41	0.73
AN	0.03	0.32	0.80
TN	0.03	0.32	0.79
OM	0.04	0.33	0.78
TP	0.01	0.08	0.94

* *P < 0.05. Soil organic matter (SOM), total and available nitrogen (TN and AN), total and available phosphorus (TP and AP), and total and available potassium (TK and AK). Number in bold means statistically significant*.

Pearson Correlation analysis was used to determine correlations between soil properties and core phyla (Table 4). Soil pH and SOM had significant correlation with Acidobacteria (*r*^2^ = −0.66 and −0.69), Beta-Proteobacteria (*r*^2^ = 0.76 and 0.70), and Bacterioidetes (*r*^2^ = 0.58 and 0.54). Beta-Proteobacteria was the most sensitive phylum responding to changes in soil properties. Besides pH and SOM, other soil properties such as TN (*r*^2^ = 0.69), TK (*r*^2^ = −0.74), AP (*r*^2^ = −0.52), and AK (*r*^2^ = 0.85), also had significant correlations with Beta-Proteobacteria. Furthermore, AP (*r*^2^ = −0.54) had negative correlation with Actinobacteria.

**Table 4:** Pearson Correlation analysis is used to determine relationship of soil property with phylum.

**phylum**	**pH**	**SOM**	**TN**	**TP**	**TK**	**AN**	**AP**	**AK**
Acidobacteria	−0.26	−0.15	−0.07	0.04	−0.14	−0.10	0.29	−0.06
Alpha-Proteobacteria	−0.66^**^	−0.69^**^	−0.23	0.37	0.39	−0.39	0.48	−0.43
Planctomycetes	0.11	0.19	0.06	0.03	−0.24	0.12	−0.33	0.21
Beta-Proteobacteria	0.76^**^	0.70^**^	0.69^**^	−0.45	−0.74^**^	0.46	−0.52^*^	0.85^***^
Actinobacteria	0.25	0.26	−0.22	0.03	0.00	−0.19	−0.54^*^	−0.10
Firmicutes	0.40	0.36	0.39	−0.43	−0.30	0.30	−0.12	0.41
Deltaproteobacteria	−0.25	−0.32	−0.27	0.28	0.16	−0.37	0.15	−0.19
Chloroflexi	0.01	0.04	−0.21	0.07	0.17	−0.01	−0.21	−0.30
Bacteroidetes	0.58^*^	0.54^*^	0.39	−0.43	−0.31	0.39	−0.35	0.49
Nitrospirae	0.37	0.25	−0.09	0.09	0.00	−0.12	−0.68	−0.02
Gemmatimonadetes	0.37	0.41	0.19	−0.098	−0.32	0.26	−0.49	0.19

* *P < 0.05*;

** *P < 0.01*;

*** *P < 0.0001. Soil organic matter (SOM), total and available nitrogen (TN and AN), total and available phosphorus (TP and AP), and total and available potassium (TK and AK). Number in bold means statistically significant*.

## Discussion

Karst rocky desertification poses as a potential threat to soil quality and ecosystem. Causes and consequences of KRD on the aboveground communities are better understood compared to those of on the underground communities, such as soil microbiome community. This study characterizes change in soil bacterial community in accordance to KRD progression, determines relationship of soil properties with bacterial community, and discusses potential activities of several bacteria in KRD regions. This study contributes to our understanding of interactions between bacterial community and KRD progression.

### Change in soil properties along KRD gradient

In this study, we find that soil pH, SOM, TN, and AK significantly increase in SKRD compared to those of in NKRD areas; whereas TP and TK significantly decrease in SKRD regions. Significant increased soil pH is one of the most common indicators in karst desertification regions (Xie et al., 2015). The high soil pH in SKRD is mainly due to soil erosion and limestone dissolving. It is worth mentioning that higher SOM content alone does not indicate higher soil quality. The significantly increased SOM in SKRD areas might be resulted from excessively dissolved limestone (Zhong et al., 2006). Besides, previous studies report that the soil stability and water accessibility are low in SKRD areas, which indicate low soil quality (Peng and Wang, 2012; Qi et al., 2017). The increased TN in SKRD soils might because (1) poor vegetation coverage; and (2) predominance of nitrogen-fixing bacteria, such as *Rhizohium* and *Frankia*. Other macronutrients, TP and TK, were significant lower in SKRD than those of in NKRD areas, indicating nutrient limiting condition in SKRD soils. Interestingly, AK was significantly higher in degraded soils, which might due to the predominance and contributions of KSB, such as *Pseudomonas, Bacillus*, and *Burkholderia* (Ahmad et al., 2016). Overall, we find that soil quality in SKRD region significantly decreased compared to that of in NKRD area.

### Soil pH primarily correlates with soil bacterial community

We find that soil pH is the main factor that correlates with soil bacterial community. Our results agree with numerous previous studies (Lauber et al., 2009; Rousk et al., 2010a,b; Yun et al., 2016). The overall close relationship of pH with bacterial community is due to most of bacteria having rather narrow pH optima (Charokopos et al., 2010). Slight change in pH affects more than half of bacterial activities (Fernández-Calviño and Bååth, 2010). The significant change in soil pH in this study is likely due to change in bacterial community composition. Beside direct impact, indirect effect of pH on soil bacterial community might also play an important role in shaping bacterial community. Soil pH has wide association with soil nutrient availability, catabolic activities, soil structure, and biomass activities (Wakelin et al., 2008). Lauber et al. proposed that change in soil pH altered soil fertility, structure, and vegetation community, thereby indirectly affecting soil bacterial community (Lauber et al., 2009). The broad interactions of soil pH with soil quality and habitat both directly and indirectly might explain the large impact of pH on soil bacterial community.

Many studies report responses of specific bacteria to soil pH gradient (Sait et al., 2006; Nicol et al., 2008; Jones et al., 2009). In this study, we find that soil pH significantly correlated with several core phyla (Acidobacteria, Beta-Proteobacteria, and Bacteroidetes). The correlation of soil pH with these bacteria is also reported in previous studies (Lauber et al., 2009; Yun et al., 2016). For instance, subgroups in Acidobacteria show varying preferences to soil pH. Relative abundances of subgroups 1 and 2 decrease in high pH; whereas those in subgroups 4, 6, 7, and 16 increase in high pH (Jones et al., 2009). Studies show that many bacterial activities, such as ammonia oxidization and phosphate solubilization, are pH-dependent (Nautiyal et al., 2000; Hu et al., 2013; Sharma et al., 2013). Phyla Acidobacteria, Beta-Proteobacteria, and Bacteroidetes contain many taxa carrying physiological functions that are pH-dependent, which might explain the large impact of pH has on those bacteria. Altogether, our results corroborate with previous studies, demonstrating that soil pH has a large impact on bacterial community.

### Correlation of other soil properties with bacterial community

Besides soil pH, other soil properties should also be considered to fully understand the relationship of environmental factors with bacterial community. Interestingly, we find that soil pH and SOM show similar correlation with core microbiome (Acidobacteria, Beta-Proteobacteria, and Bacteroidetes), suggesting synergetic effect of pH and SOM on soil bacterial community. Indeed, a previous study reports significant correlation between soil pH and microbial decomposition rate of SOM (Yun et al., 2016). Change in soil pH affects bacterial efficiency of catabolizing cysteine, aspartic acid, lysine, and arginine (Wakelin et al., 2008). The highly alkaline soil in SKRD area results in low SOM decomposition rate, which also explain the accumulated SOM content in SKRD (Leahy and Colwell, 1990).

One member of the core microbiome, Beta-Proteobacteria, is highly responsive to change in soil properties. Six out of eight soil properties (pH, SOM, TN, TK, AP, and AK) show significant correlation with Beta-Proteobacteria. Beta-Proteobacteria contains many taxa that are highly efficient on mineral weathering in soils (Lepleux et al., 2012). Mineral weathering releases nutritive cations (e.g., phosphate, iron, and granite), which are particularly essential in nutrient limiting soils (Uroz et al., 2011). Although the result was not significant, Beta-Proteobacteria was the most abundant in M- and S-KRD soils (Figure 2). The predominance of Beta-Proteobacteria in degraded soils suggests potential nutrient releasing and cycling functions. Many taxa that belong to *Bacillus* (Beta-Proteobacteria) have high efficacy in mineral dissolving (Song et al., 2007; Uroz et al., 2011). The overall change in *Bacillus* increased along with KRD progression (Figure 4). The relatively high abundance of *Bacillus* further implies nutrient releasing activities in KRD area. Soil nutrient composition affects bacterial community structure (Uroz et al., 2009). For instance, various carbon sources (xylose, glucose, lactose, and mannitol) and nitrogen sources (nitrate, ammonium, and mixture of nitrate and ammonium) affect bacterial physiological activities (Nautiyal et al., 2000; Hameeda et al., 2006). The high sensitivity of Beta-Proteobacteria to change in soil properties might be explained as (1) the mineral weathering is dependent on nutrient availability in soils and (2) significant changes in soil chemical properties have large impact on Beta-Proteobacteria.

We find that phylum Cyanobacteria significantly predominate in NKRD soils. Cyanobacteria, along with lichens and mosses, compose biological soil crusts (BSCs), which preserve soil fertility in dry land (Coxson, 2002). Studies show that filamentous Cyanobacteria, such as *Nostoc* and *Leptolyngbya*, improve soil quality by providing nutrients and stabilizing soil structure (Büdel et al., 2009; Maqubela et al., 2009). Cyanobacteria provide carbon and nitrogen compounds via C and N fixing in soils (Housman et al., 2007). Cyanobacteria also produce polysaccharides that form extracellular polymeric substances (EPS) to coat and stabilize soil aggregates (Aspiras et al., 1971). The significant abundance of Cyanobacteria in NKRD regions might partially explain the better soil quality in non-degraded soils, and vice versa.

Overall, we find that soil bacterial community compositions in degraded soils are distinguishingly different from those of in non-degraded soils. Soil pH primarily correlates with soil microbiome. Other soil properties, such as SOM, TN, TK, AP, and AK also have correlation with soil bacterial composition. Cyanobacteria, which have high potential of improving soil quality, is significantly abundant in NKRD. Many taxa with mineral weathering and nutrient cycling potentials predominate in degraded soils, suggesting that soil bacteria are important in providing nutrients in barren soils. This study fills a gap of knowledge on relationship of soil properties with bacterial community in KRD areas.

## Author contributions

XW, DQ, and UD designed experiments. DQ and XZ collected and measured samples. XW analyzed data and wrote the manuscript. XW and UD edited the manuscript.

### Conflict of interest statement

The authors declare that the research was conducted in the absence of any commercial or financial relationships that could be construed as a potential conflict of interest.

## Supplementary material

Supplementary Figure 1 Change in Alpha-Proteobacteria **(A)** and Beta-Proteobacteria **(B)** along with karst rocky desertification gradient. No KRD (NKRD) is in red color, latent KRD (LKRD) is in green color, moderate KRD (MKRD) is in yellow color, and severe KRD (SKRD) is in purple color.

Supplementary Figure 2 Change in diversity (Shannon index) **(A)** and richness (Ace) **(B)** along with karst rocky desertification gradient. No KRD (NKRD) is in red color, latent KRD (LKRD) is in green color, moderate KRD (MKRD) is in yellow color, and severe KRD (SKRD) is in purple color.

Supplementary Table 1 Soil properties in karst rocky desertification areas. No KRD (NKRD), latent KRD (LKRD), moderate KRD (MKRD), and severe KRD (SKRD). Soil organic matter (SOM), total and available nitrogen (TN and AN), total and available phosphorus (TP and AP), and total and available potassium (TK and AK).

Supplementary Table 2 One-way ANOVA results of soil properties. One-way Analysis of variance (One-way ANOVA) analysis. Variable is significantly different if *P*-value < 0.05. *P*-value is in bold if the difference is significant. Soil organic matter (SOM), total and available nitrogen (TN and AN), total and available phosphorus (TP and AP), and total and available potassium (TK and AK).

Supplementary Table 3 OTU classification at phylum, class, order, family, and genus level. No KRD (NKRD), latent KRD (LKRD), moderate KRD (MKRD), and severe KRD (SKRD).

Supplementary Table 4 One-way ANOVA results of phyla. One-way Analysis of variance (One-way ANOVA) analysis. Variable is significantly different if *P*-value < 0.05. *P*-value is in bold if the difference is significant.

Supplementary Table 5 Average reads classified in Alpha- and Beta-Proteobacteria in karst rocky desertification regions. The Tukey's HSD test is used to determine the significant difference between variables. Different letters are assigned to significant difference, *P* < 0.05. No KRD (NKRD), latent KRD (LKRD), moderate KRD (MKRD), and severe KRD (SKRD).

## References

[B1] Ahmad M. Nadeem S. M. Naveed M. Zahir Z. A. (2016). Potassium-solubilizing bacteria and their application in agriculture, in Potassium Solubilizing Microorganisms for Sustainable Agriculture, eds Meena V. S. Maurya B. R. Verma J. P. Meena R. S. (New Delhi: Springer), 293–313.

[B2] Aspiras R. Allen O. Chesters G. Harris R. (1971). Chemical and physical stability of microbially stabilized aggregates. Soil. Sci. Soc. Am. J. 35, 283–286. 10.2136/sssaj1971.03615995003500020030x

[B3] Büdel B. Darienko T. Deutschewitz K. Dojani S. Friedl T. Mohr K. I.. (2009). Southern african biological soil crusts are ubiquitous and highly diverse in drylands, being restricted by rainfall frequency. Microb. Ecol. 57, 229–247. 10.1007/s00248-008-9449-9 18850242

[B4] Calò F. Parise M. (2006). Evaluating the human disturbance to karst environments in southern Italy. Acta Carsologica 35, 47–56. 10.3986/ac.v35i2-3.227

[B5] Chaparro J. M. Sheflin A. M. Manter D. K. Vivanco J. M. (2012). Manipulating the soil microbiome to increase soil health and plant fertility. Biol. Fertil. Soils 48, 489–499. 10.1007/s00374-012-0691-4

[B6] Charokopos N. Artemiou P. Antonitsis P. Rouska E. (2010). Repair of aortic regurgitation caused by spontaneous avulsion of aortic valve commissure in a patient with idiopathic thrombocytopenic purpura. Thorac. Cardiovasc. Surg. 58, 43–44. 10.1055/s-2008-1039057 20072976

[B7] Coxson D. (2002). Biological soil crusts: structure, function, and management. Bryologist 105, 500–501.

[B8] De'Ath G. (2002). Multivariate regression trees: a new technique for modeling species–environment relationships. Ecology 83, 1105–1117. 10.1890/0012-9658(2002)083[1105:MRTANT]2.0.CO;2

[B9] De Boer W. Kowalchuk G. (2001). Nitrification in acid soils: micro-organisms and mechanisms. Soil Biol. Biochem. 33, 853–866. 10.1016/S0038-0717(00)00247-9

[B10] Edgar R. C. Haas B. J. Clemente J. C. Quince C. Knight R. (2011). UCHIME improves sensitivity and speed of chimera detection. Bioinformatics 27, 2194–2200. 10.1093/bioinformatics/btr381 21700674 PMC3150044

[B11] Fernández-Calviño D. Bååth E. (2010). Growth response of the bacterial community to pH in soils differing in pH. FEMS Microbiol. Ecol. 73, 149–156. 10.1111/j.1574-6941.2010.00873.x 20455934

[B12] Fierer N. Bradford M. A. Jackson R. B. (2007). Toward an ecological classification of soil bacteria. Ecology 88, 1354–1364. 10.1890/05-1839 17601128

[B13] Ford D. C. Williams P. W. (1989). Karst Geomorphology and Hydrology. London: Unwin Hyman.

[B14] Gong Z. (2001). Chinese Soil Taxonomy. Beijing: Science press.

[B15] Gutiérrez F. Parise M. De Waele J. Jourde H. (2014). A review on natural and human-induced geohazards and impacts in karst. Earth Sci. Rev. 138, 61–88. 10.1016/j.earscirev.2014.08.002

[B16] Hameeda B. Reddy Y. H. Rupela O. P. Kumar G. N. Reddy G. (2006). Effect of carbon substrates on rock phosphate solubilization by bacteria from composts and macrofauna. Curr. Microbiol. 53, 298–302. 10.1007/s00284-006-0004-y 16941242

[B17] Housman D. C. Yeager C. M. Darby B. J. Sanford R. L. Kuske C. R. Neher D. A. (2007). Heterogeneity of soil nutrients and subsurface biota in a dryland ecosystem. Soil Biol. Biochem. 39, 2138–2149. 10.1016/j.soilbio.2007.03.015

[B18] Hu H.-W. Zhang L.-M. Dai Y. Di H.-J. He J.-Z. (2013). pH-dependent distribution of soil ammonia oxidizers across a large geographical scale as revealed by high-throughput pyrosequencing. J. Soil Sediment. 13, 1439–1449. 10.1007/s11368-013-0726-y

[B19] Jiang Z. Lian Y. Qin X. (2014). Rocky desertification in Southwest China: impacts, causes, and restoration. Earth Sci. Rev. 132, 1–12. 10.1016/j.earscirev.2014.01.005

[B20] Jones R. T. Robeson M. S. Lauber C. L. Hamady M. Knight R. Fierer N. (2009). A comprehensive survey of soil acidobacterial diversity using pyrosequencing and clone library analyses. ISME J. 3, 442–453. 10.1038/ismej.2008.127 19129864 PMC2997719

[B21] Karlen D. L. Mausbach M. J. Doran J. W. Cline R. G. Harris R. F. Schuman G. E. (1997). Soil quality: a concept, definition, and framework for evaluation (a guest editorial). Soil Sci. Soc. Am. J. 61, 4–10. 10.2136/sssaj1997.03615995006100010001x

[B22] Kowalchuk G. A. Stephen J. R. (2001). Ammonia-oxidizing bacteria: a model for molecular microbial ecology. Annu. Rev. Microbiol. 55, 485–529. 10.1146/annurev.micro.55.1.485 11544365

[B23] Lakshmanan V. Selvaraj G. Bais H. P. (2014). Functional soil microbiome: belowground solutions to an aboveground problem. Plant Physiol. 166, 689–700. 10.1104/pp.114.245811 25059708 PMC4213098

[B24] Lauber C. L. Hamady M. Knight R. Fierer N. (2009). Pyrosequencing-based assessment of soil pH as a predictor of soil bacterial community structure at the continental scale. Appl. Environ. Microbiol. 75, 5111–5120. 10.1128/AEM.00335-09 19502440 PMC2725504

[B25] Leahy J. G. Colwell R. R. (1990). Microbial degradation of hydrocarbons in the environment. Microbiol. Rev. 54, 305–315. 2215423 10.1128/mr.54.3.305-315.1990 PMC372779

[B26] Lepleux C. Turpault M. P. Oger P. Frey-Klett P. Uroz S. (2012). Correlation of the abundance of betaproteobacteria on mineral surfaces with mineral weathering in forest soils. Appl. Environ. Microb. 78, 7114–7119. 10.1128/AEM.00996-12 22798365 PMC3457497

[B27] Li Y.- B. Shao J.-A. Yang H. Bai X.-Y. (2009). The relations between land use and karst rocky desertification in a typical karst area, China. Environ. Geol. 57, 621–627. 10.1007/s00254-008-1331-z

[B28] Li Y. Shao M. (2006). Change of soil physical properties under long-term natural vegetation restoration in the Loess Plateau of China. J. Arid. Environ. 64, 77–96. 10.1016/j.jaridenv.2005.04.005

[B29] Lozupone C. Lladser M. E. Knights D. Stombaugh J. Knight R. (2011). UniFrac: an effective distance metric for microbial community comparison. ISME J. 5:169. 10.1038/ismej.2010.133 20827291 PMC3105689

[B30] Maqubela M. Mnkeni P. Issa O. M. Pardo M. D'Acqui L. (2009). Nostoc cyanobacterial inoculation in South African agricultural soils enhances soil structure, fertility, and maize growth. Plant Soil 315, 79–92. 10.1007/s11104-008-9734-x

[B31] Mursyida E. Mubarik N. R. Tjahjoleksono A. (2015). Selection and identification of phosphate-potassium solubilizing bacteria from the area around the limestone mining in Cirebon quarry. Res. J. Microbiol. 10, 270–279. 10.3923/jm.2015.270.279

[B32] Nautiyal C. S. Bhadauria S. Kumar P. Lal H. Mondal R. Verma D. (2000). Stress induced phosphate solubilization in bacteria isolated from alkaline soils. FEMS Microbiol. Lett. 182, 291–296. 10.1111/j.1574-6968.2000.tb08910.x 10620681

[B33] Nelson D. W. Sommers L. E. (1982). Total carbon, organic carbon, and organic matter, in Methods of Soil Analysis: Chemical and Microbiological Properties, eds Page A. L. Millerr H. Keeney D. R. (Madison, WI: American Society of Agronomy), 539–552.

[B34] Nicol G. W. Leininger S. Schleper C. Prosser J. I. (2008). The influence of soil pH on the diversity, abundance and transcriptional activity of ammonia oxidizing archaea and bacteria. Environ. Microbiol. 10, 2966–2978. 10.1111/j.1462-2920.2008.01701.x 18707610

[B35] Oksanen J. Blanchet F. Kindt R. Legendre P. Minchin P. O'Hara R. (2014). Vegan: Community Ecology Package. R package version 2.1-41/r2867.

[B36] Peng J. Xu Y. Cai Y. Xiao H. (2011). Climatic and anthropogenic drivers of land use/cover change in fragile karst areas of southwest China since the early 1970s: a case study on the Maotiaohe watershed. Environ. Earth Sci. 64, 2107–2118. 10.1007/s12665-011-1037-5

[B37] Peng T. Wang S.-J. (2012). Effects of land use, land cover and rainfall regimes on the surface runoff and soil loss on karst slopes in southwest China. Catena 90, 53–62. 10.1016/j.catena.2011.11.001

[B38] Qi D. Wieneke X. Zhou X. Jiang X. Xue P. (2017). Succession of vegetation community composition and leaf functional traits in responding to karst rocky desertification in the Wushan County in Chongqing, China. Commun. Ecol. 18, 157–168. 10.1556/168.2017.18.2.5

[B39] Quast C. Pruesse E. Yilmaz P. Gerken J. Schweer T. Yarza P.. (2013). The SILVA ribosomal RNA gene database project: improved data processing and web-based tools. Nucleic Acids Res. 41, D590–D596. 10.1093/nar/gks1219 23193283 PMC3531112

[B40] Rime T. Hartmann M. Brunner I. Widmer F. Zeyer J. Frey B. (2015). Vertical distribution of the soil microbiota along a successional gradient in a glacier forefield. Mol. Ecol. 24, 1091–1108. 10.1111/mec.13051 25533315

[B41] Rousk J. Bååth E. Brookes P. C. Lauber C. L. Lozupone C. Caporaso J. G.. (2010a). Soil bacterial and fungal communities across a pH gradient in an arable soil. ISME J. 4, 1340–1351. 10.1038/ismej.2010.58 20445636

[B42] Rousk J. Brookes P. C. Baath E. (2010b). Investigating the mechanisms for the opposing pH relationships of fungal and bacterial growth in soil. Soil Biol. Biochem. 42, 926–934. 10.1016/j.soilbio.2010.02.009

[B43] Sait M. Davis K. E. Janssen P. H. (2006). Effect of pH on isolation and distribution of members of subdivision 1 of the phylum Acidobacteria occurring in soil. Appl. Environ. Microbiol. 72, 1852–1857. 10.1128/AEM.72.3.1852-1857.2006 16517631 PMC1393200

[B44] Schloss P. D. Westcott S. L. Ryabin T. Hall J. R. Hartmann M. Hollister E. B.. (2009). Introducing mothur: open-source, platform-independent, community-supported software for describing and comparing microbial communities. Appl. Environ. Microbiol. 75, 7537–7541. 10.1128/AEM.01541-09 19801464 PMC2786419

[B45] Segata N. Izard J. Waldron L. Gevers D. Miropolsky L. Garrett W. S.. (2011). Metagenomic biomarker discovery and explanation. Genome Biol. 12:R60. 10.1186/gb-2011-12-6-r60 21702898 PMC3218848

[B46] Shah V. Shah S. Kambhampati M. S. Ambrose J. Smith N. Dowd S. E.. (2011). Bacterial and archaea community present in the Pine Barrens Forest of Long Island, NY: unusually high percentage of ammonia oxidizing bacteria. PLoS ONE 6:e26263. 10.1371/journal.pone.0026263 22028845 PMC3197628

[B47] Sharma S. B. Sayyed R. Z. Trivedi M. H. Gobi T. A. (2013). Phosphate solubilizing microbes: sustainable approach for managing phosphorus deficiency in agricultural soils. Springerplus 2:587. 10.1186/2193-1801-2-587 25674415 PMC4320215

[B48] Song W. Ogawa N. Oguchi C. Hatta T. Matsukura Y. (2007). Effect of Bacillus subtilis on granite weathering: a laboratory experiment. Catena 70, 275–281. 10.1016/j.catena.2006.09.003

[B49] Team R. C. (2013). R: A Language and Environment for Statistical Computing. Vienna: R Foundation for Statistical Computing Available online at: URL http://www.R-project.org/

[B50] Uroz S. Calvaruso C. Turpault M.-P. Frey-Klett P. (2009). Mineral weathering by bacteria: ecology, actors and mechanisms. Trends Microbiol. 17, 378–387. 10.1016/j.tim.2009.05.004 19660952

[B51] Uroz S. Oger P. Lepleux C. Collignon C. Frey-Klett P. Turpault M.-P. (2011). Bacterial weathering and its contribution to nutrient cycling in temperate forest ecosystems. Res. Microbiol. 162, 820–831. 10.1016/j.resmic.2011.01.013 21315149

[B52] Wakelin S. A. Macdonald L. M. Rogers S. L. Gregg A. L. Bolger T. P. Baldock J. A. (2008). Habitat selective factors influencing the structural composition and functional capacity of microbial communities in agricultural soils. Soil Biol. Biochem. 40, 803–813. 10.1016/j.soilbio.2007.10.015

[B53] Walkley A. Black I. A. (1934). An examination of the Degtjarrtt method for determining soil organic matter and proposed modification of chromic acid titration method. Soil Sci. 37, 29–38. 10.1097/00010694-193401000-00003

[B54] Wang S. J. Liu Q. M. Zhang D. F. (2004). Karst rocky desertification in southwestern China: geomorphology, land use, impact and rehabilitation. Land Degrad. Dev. 15, 115–121. 10.1002/ldr.592

[B55] Xie L. Zhong J. Chen F. Cao F. Li J. Wu L. (2015). Evaluation of soil fertility in the succession of karst rocky desertification using principal component analysis. Solid Earth 6:515 10.5194/se-6-515-2015

[B56] Yan X. Cai Y. (2015). Multi-scale anthropogenic driving forces of karst rocky desertification in southwest china. Land Degrad. Dev. 26, 193–200. 10.1002/ldr.2209

[B57] Yao H. He Z. Wilson M. J. Campbell C. D. (2000). Microbial biomass and community structure in a sequence of soils with increasing fertility and changing land use. Microbial. Ecol. 40, 223–237. 10.1007/s002480000053 11080380

[B58] Yun Y. Wang H. Man B. Xiang X. Zhou J. Qiu X.. (2016). The relationship between pH and bacterial communities in a single karst ecosystem and its implication for soil acidification. Front. Microbiol. 7:1955. 10.3389/fmicb.2016.01955 28018299 PMC5159436

[B59] Zeng F. Peng W. Song T. Wang K. Wu H. Song X. (2007). Changes in vegetation after 22 years' natural restoration in the Karst disturbed area in northwestern Guangxi, China. Acta Ecologica Sinica 27, 5110–5119. 10.1016/S1872-2032(08)60016-5

[B60] Zhang J. Y. Dai M. H. Wang L. C. Zeng C. F. Su W. C. (2016). The challenge and future of rocky desertification control in karst areas in southwest China. Solid Earth 7, 83–91. 10.5194/se-7-83-2016

[B61] Zhong Y. Tang J. Wang L. (2006). Distribution characteristic of soil organic carbon in Three Gorges Reservoir District. J. Soil Water Conserv. 20, 73–76. 10.13870/j.cnki.stbcxb.2006.05.018

[B62] Zolla G. Badri D. V. Bakker M. G. Manter D. K. Vivanco J. M. (2013). Soil microbiomes vary in their ability to confer drought tolerance to Arabidopsis. Appl. Soil Ecol. 68, 1–9 10.1016/j.apsoil.2013.03.007

