# Supplementary figures and images for: Soil pH Is the Primary Factor Correlating With Soil Microbiome in Karst Rocky Desertification Regions in the Wushan County, Chongqing, China
Source: Front Microbiol. 2018 May 29;9:1027. doi: 10.3389/fmicb.2018.01027 (PMC5987757; doi:10.3389/fmicb.2018.01027)

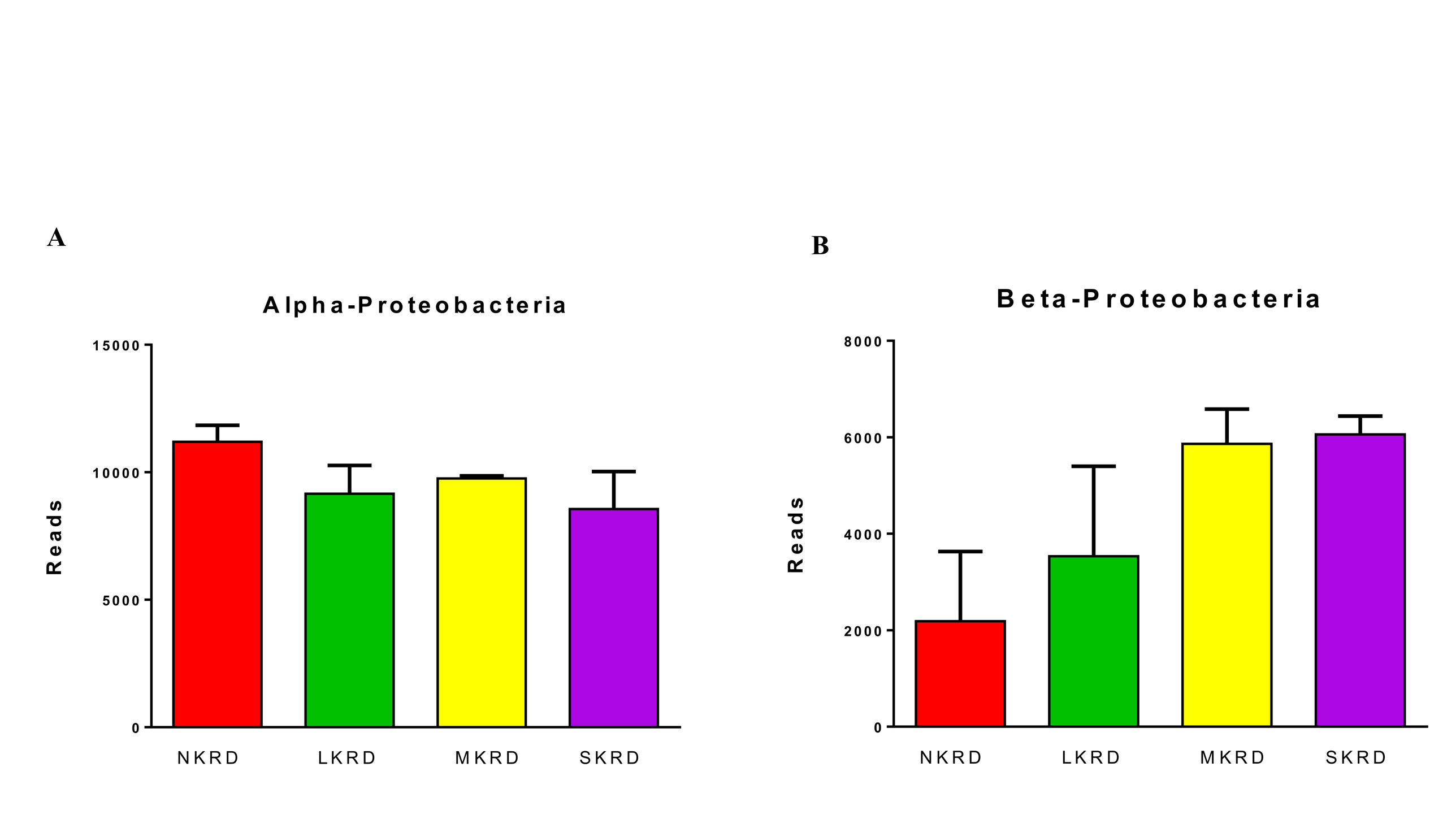

Supplement: Supplementary Figure 1 — Change in Alpha-Proteobacteria (A) and Beta-Proteobacteria (B) along with karst rocky desertification gradient. No KRD (NKRD) is in red color, latent KRD (LKRD) is in green color, moderate KRD (MKRD) is in yellow color, and severe KRD (SKRD) is in purple color. [file Image_1.tif]

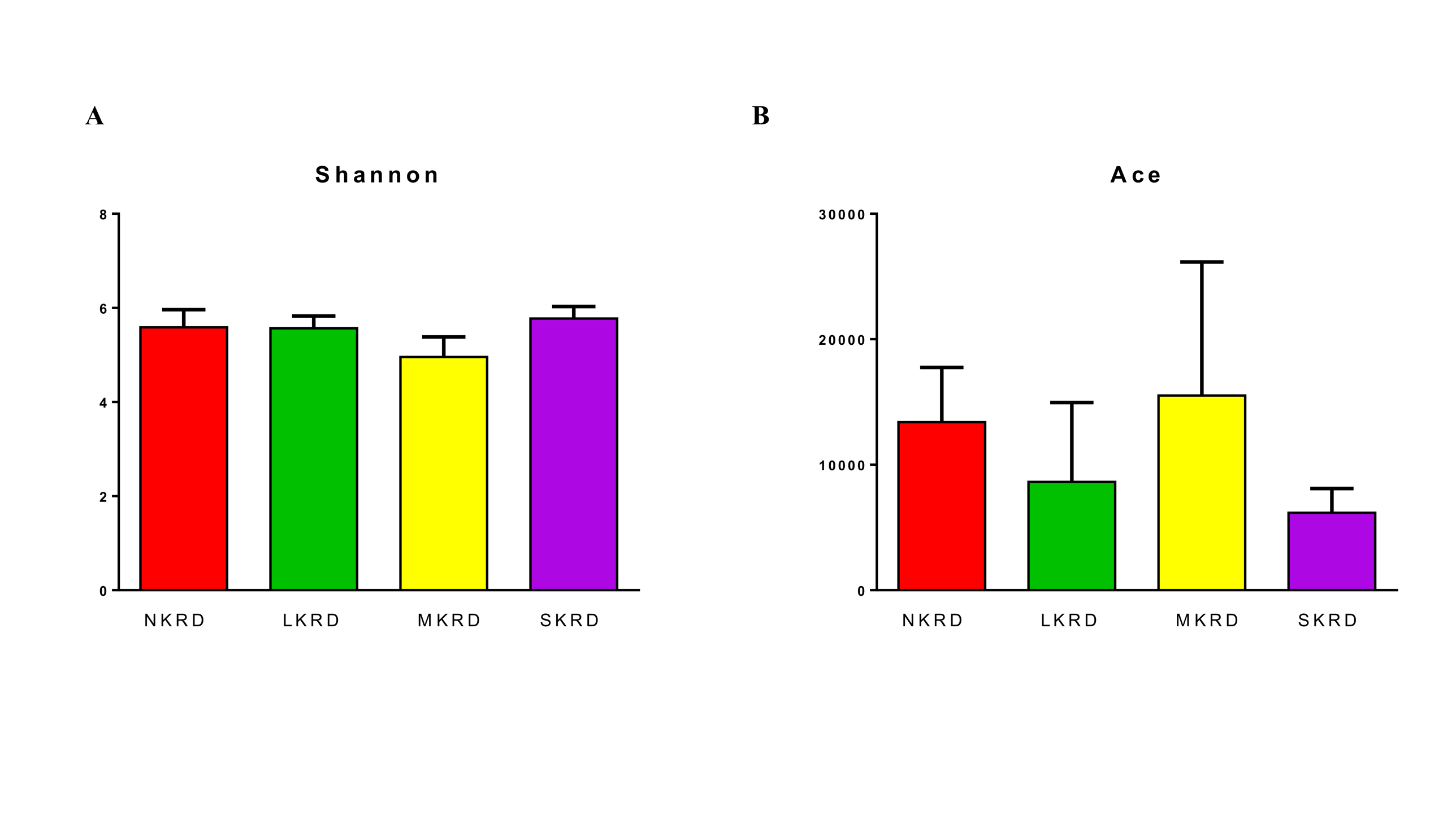

Supplement: Supplementary Figure 2 — Change in diversity (Shannon index) (A) and richness (Ace) (B) along with karst rocky desertification gradient. No KRD (NKRD) is in red color, latent KRD (LKRD) is in green color, moderate KRD (MKRD) is in yellow color, and severe KRD (SKRD) is in purple color. [file Image_2.tif]
