# Supplementary material for: Soil pH Is the Primary Factor Correlating With Soil Microbiome in Karst Rocky Desertification Regions in the Wushan County, Chongqing, China
Source: Front Microbiol. 2018 May 29;9:1027. doi: 10.3389/fmicb.2018.01027 (PMC5987757; doi:10.3389/fmicb.2018.01027)
Supplement: Supplementary Table 1 — Soil properties in karst rocky desertification areas. No KRD (NKRD), latent KRD (LKRD), moderate KRD (MKRD), and severe KRD (SKRD). Soil organic matter (SOM), total and available nitrogen (TN and AN), total and available phosphorus (TP and AP), and total and available potassium (TK and AK). [file Table_1.DOCX]

**Supplementary Table 1** Soil properties in karst rocky desertification areas.

|  | pH | OM g/kg | TN g/kg | TP g/kg | TK g/kg | AN mg/g | AP mg/g | AK mg/g |
| --- | --- | --- | --- | --- | --- | --- | --- | --- |
| NKRD_1 | 5.96 | 8.62 | 0.35 | 0.35 | 12.56 | 32.74 | 2.5 | 135.82 |
| NKRD_2 | 6.18 | 16.81 | 0.48 | 0.31 | 14.51 | 58.37 | 2.84 | 135.75 |
| NKRD_3 | 6.07 | 5.66 | 0.38 | 0.29 | 15.76 | 44.97 | 3.76 | 137.8 |
| LKRD_1 | 7.55 | 20.08 | 0.55 | 0.27 | 14.73 | 50.57 | 2.09 | 150.45 |
| LKRD_2 | 7.81 | 34.36 | 0.38 | 0.28 | 14.03 | 50.00 | 2.17 | 142.33 |
| LKRD_3 | 7.76 | 28.44 | 0.35 | 0.34 | 15.49 | 42.74 | 2.03 | 146.55 |
| MKRD_1 | 7.96 | 43.98 | 0.55 | 0.20 | 10.54 | 65.57 | 2.41 | 181.83 |
| MKRD_2 | 8.07 | 32.14 | 0.63 | 0.34 | 11.58 | 58.26 | 2.28 | 178.00 |
| MKRD_3 | 8.13 | 41.54 | 0.65 | 0.18 | 10.03 | 63.99 | 2.63 | 186.67 |
| SKRD_1 | 8.15 | 48.34 | 0.63 | 0.20 | 10.01 | 72.26 | 2.4.0 | 176.33 |
| SKRD_2 | 8.82 | 47.99 | 0.65 | 0.18 | 10.71 | 59.49 | 2.07 | 182.00 |
| SKRD_3 | 8.61 | 46.07 | 0.73 | 0.16 | 9.26 | 66.17 | 2.39 | 178.33 |

No KRD (NKRD), latent KRD (LKRD), moderate KRD (MKRD), and severe KRD (SKRD). Soil organic matter (SOM), total and available nitrogen (TN and AN), total and available phosphorus (TP and AP), and total and available potassium (TK and AK).
