# Supplementary material for: Soil pH Is the Primary Factor Correlating With Soil Microbiome in Karst Rocky Desertification Regions in the Wushan County, Chongqing, China
Source: Front Microbiol. 2018 May 29;9:1027. doi: 10.3389/fmicb.2018.01027 (PMC5987757; doi:10.3389/fmicb.2018.01027)
Supplement: Supplementary Table 2 — One-way ANOVA results of soil properties. One-way Analysis of variance (One-way ANOVA) analysis. Variable is significantly different if P-value < 0.05. P-value is in bold if the difference is significant. Soil organic matter (SOM), total and available nitrogen (TN and AN), total and available phosphorus (TP and AP), and total and available potassium (TK and AK). [file Table_2.DOCX]

**Supplementary Table 2** One-way ANOVA results of soil properties.

|  | | SS | df | MS | F | *P* value |
| --- | --- | --- | --- | --- | --- | --- |
| pH | Treatment | 10 | 3 | 3.40 | 88 | **<0.0001** |
|  | Residual | 0.31 | 6 | 0.04 |  |  |
|  | Total | 11 | 11 |  |  |  |
| SOM | Treatment | 2328 | 3 | 776 | 20 | **0.03** |
|  | Residual | 234 | 6 | 39 |  |  |
|  | Total | 2578 | 11 |  |  |  |
| TN | Treatment | 0.16 | 3 | 0.05 | 7.30 | 0.07 |
|  | Residual | 0.04 | 6 | 0.007 |  |  |
|  | Total | 0.20 | 11 |  |  |  |
| TP | Treatment | 0.03 | 3 | 0.01 | 3.70 | 0.14 |
|  | Residual | 0.02 | 6 | 0.003 |  |  |
|  | Total | 0.06 | 11 |  |  |  |
| TK | Treatment | 53 | 3 | 18 | 15 | **0.03** |
|  | Residual | 7.20 | 6 | 1.20 |  |  |
|  | Total | 62 | 11 |  |  |  |
| AN | Treatment | 968 | 3 | 323 | 4.10 | 0.16 |
|  | Residual | 469 | 6 | 78 |  |  |
|  | Total | 1446 | 11 |  |  |  |
| AP | Treatment | 1.50 | 3 | 0.49 | 4.50 | 0.15 |
|  | Residual | 0.65 | 6 | 0.11 |  |  |
|  | Total | 2.50 | 11 |  |  |  |
| AK | Treatment | 4747 | 3 | 1582 | 128 | **0.0009** |
|  | Residual | 74 | 6 | 12 |  |  |
|  | Total | 4837 | 11 |  |  |  |

One-way Analysis of variance (One-way ANOVA) analysis. Variable is significantly different if *P* value < 0.05. *P* value is in bold if the difference is significant. Soil organic matter (SOM), total and available nitrogen (TN and AN), total and available phosphorus (TP and AP), and total and available potassium (TK and AK).
