# Supplementary material for: Soil pH Is the Primary Factor Correlating With Soil Microbiome in Karst Rocky Desertification Regions in the Wushan County, Chongqing, China
Source: Front Microbiol. 2018 May 29;9:1027. doi: 10.3389/fmicb.2018.01027 (PMC5987757; doi:10.3389/fmicb.2018.01027)
Supplement: Supplementary Table 4 — One-way ANOVA results of phyla. One-way Analysis of variance (One-way ANOVA) analysis. Variable is significantly different if P-value < 0.05. P-value is in bold if the difference is significant. [file Table_4.docx]

**Supplementary Table 4** One-way ANOVA results of phyla.

|  | | SS | df | MS | F | *P* value |
| --- | --- | --- | --- | --- | --- | --- |
| Alpha-Proteobacteria | Treatment | 1.4e+008 | 3 | 4.8e+007 | 8 | **0.009** |
|  | Residual | 4.8e+007 | 8 | 6.0e+006 |  |  |
|  | Total | 1.9e+008 | 11 |  |  |  |
| Beta-Proteobacteria | Treatment | 3.2e+007 | 3 | 1.1e+007 | 6.8 | **0.01** |
|  | Residual | 1.2e+007 | 8 | 1.6e+006 |  |  |
|  | Total | 4.4e+007 | 11 |  |  |  |

One-way Analysis of variance (One-way ANOVA) analysis. Variable is significantly different if *P* value < 0.05. *P* value is in bold if the difference is significant.
