# Supplementary material for: Soil pH Is the Primary Factor Correlating With Soil Microbiome in Karst Rocky Desertification Regions in the Wushan County, Chongqing, China
Source: Front Microbiol. 2018 May 29;9:1027. doi: 10.3389/fmicb.2018.01027 (PMC5987757; doi:10.3389/fmicb.2018.01027)
Supplement: Supplementary Table 5 — Average reads classified in Alpha- and Beta-Proteobacteria in karst rocky desertification regions. The Tukey's HSD test is used to determine the significant difference between variables. Different letters are assigned to significant difference, P < 0.05. No KRD (NKRD), latent KRD (LKRD), moderate KRD (MKRD), and severe KRD (SKRD). [file Table_5.docx]

**Supplementary Table 5.** Average reads classified in Alpha- and Beta-Proteobacteria in karst rocky desertification regions

|  | Alpha-Proteobacteria | Beta-Proteobacteria |
| --- | --- | --- |
| NKRD | 11191a | 2188b |
| LKRD | 9160a | 3534ab |
| MKRD | 9758a | 5868a |
| SKRD | 8556b | 6060a |

The Tukey's HSD test is used to determine the significant difference between variables. Different letters are assigned to significant difference, *P*< 0.05. No KRD (NKRD), latent KRD (LKRD), moderate KRD (MKRD), and severe KRD (SKRD).
